# Factors Associated with the Number of Injured and Fatalities in Motor Vehicle Intentional Mass-Casualty Incidents: A Timely Aid for Scaling the Emergency Response

**DOI:** 10.1017/S1049023X23006726

**Published:** 2024-02

**Authors:** Eva Maria Valiño, Rafael Castro-Delgado, Silvia Sola Muñoz, Barry Lynam, Pedro Castro

**Affiliations:** 1. Emergency Medical System, Catalonia, Spain; 2. University of Barcelona, Barcelona, Spain; 3. Department of Medicine, Oviedo University, Oviedo, Spain; 4. Health Service of the Principality of Asturias (SAMU-Asturias), Health Research Institute of the Principality of Asturias (Research Group on Prehospital Care and Disasters, GIAPREDE), Oviedo, Spain; 5. RINVEMER-SEMES (Research Network on Prehospital Care-Spanish Society of Emergency Medicine), Madrid, Spain; 6. Medical Intensive Care Unit, Hospital Clinic of Barcelona; IDIBAPS; University of Barcelona, Barcelona, Spain

**Keywords:** Emergency Medical Services, mass-casualty incidents, motor vehicles, TARMAC, terrorism

## Abstract

**Introduction:**

Intentional mass-casualty incidents (IMCIs) involving motor vehicles (MVs) as weapons represent a growing trend in Western countries. This method has resulted in the highest casualty rates per incident within the field of IMCIs. Consequently, there is an urgent requirement for a timely and accurate casualty estimation in MV-induced IMCIs to scale and adjust the necessary health care resources.

**Study Objective:**

The objective of this study is to identify the factors associated with the number of casualties during the initial phase of MV-IMCIs.

**Methods:**

This is a retrospective, observational, analytical study on MV-IMCIs world-wide, from 2000-2021. Data were obtained from three different sources: Targeted Automobile Ramming Mass-Casualty Attacks (TARMAC) Attack Database, Global Terrorism Database (GTD), and the vehicle-ramming attack page from the Wikipedia website. Jacobs’ formula was used to estimate the population density in the vehicle’s route. The primary outcome variables were the total number of casualties (injured and fatalities). Associations between variables were analyzed using Spearman’s correlation coefficient and simple linear regression.

**Results:**

Forty-six MV-IMCIs resulted in 1,636 casualties (1,430 injured and 206 fatalities), most of them caused by cars. The most frequent driving pattern was accelerating whilst approaching the target, with an average speed range between four to 130km/h and a distance traveled between ten to 2,260 meters. The people estimated in the MV-IMCI scenes ranged from 36-245,717. A significant positive association was found of the number affected with the estimated crowd in the scene (R^2^: 0.64; 95% CI, 0.61-0.67; P <.001) and the average vehicle speed (R^2^: 0.42; 95% CI, 0.40-0.44; P = .004).

**Conclusion:**

The estimated number of people in the affected area and vehicle’s average speed are the most significant variables associated with the number of casualties in MV-IMCIs, helping to enable a timely estimation of the casualties.

## Introduction

An intentional mass-casualty incident (IMCI) is an event caused with the intention of generating as many victims as possible. These IMCIs create an initial imbalance between the demand for care and the available resources, increasing the mortality of those casualties.^1–4^ Furthermore, over-response to these incidents leads to a resource misallocation that can also be detrimental to non-incident patients.^4^

Most Emergency Medical Services (EMS) and hospitals decide their level of mass-casualty incident response according to the initial estimation of casualties at the scene and considering the expected percentage of seriously injured patients.^5,6^ Waiting for a complete triage to be carried out can delay the deployment of an adequate response to the incident and increase the preventable mortality. However, there are great difficulties in adequately measuring the number of casualties in this type of incident at an early stage, mainly because the information at the beginning is scarce and imprecise giving way to under-estimates as well as over-preparedness.^6–8^

Using motor vehicles (MVs) as weapons in IMCIs is a growing trend in Western countries, especially since 2016.^1,2,6,7,9–12^ A recent example occurred in the parking lot of a health center in the town of Haro, La Rioja, Spain (September 2023) where the assailant rammed a group of people causing five injuries and one death, four of them health care workers.^13^ In fact, this mechanism has been, in recent years, the one that has caused the highest number of casualties per incident in the context of IMCIs.^1^ Moreover, the injuries found in patients intentionally run over by a MV are more serious than unintentional running over, producing higher mortality and morbidity. Thus, these patients generate a high demand for both surgical and intensive care resources in the initial response phase of the incident.^2,6–8,10,14,15^

A rapid and early estimate of the number of casualties due to MV-IMCIs is therefore needed to adjust the necessary health care resources to respond both in the prehospital and in-hospital phases, when scarce information is available.^6,9,16^

The objective of this study is to identify the factors associated with the number of casualties (injured/fatalities) during the initial phase of MV-IMCIs in order to improve the estimation of the resources required.

## Methods

### Study Design and Data Source

This analytical observational study investigated MV-IMCIs world-wide, gathering and analyzing a retrospective data collection, spanning from January 2000 through December 2021. To enhance data reliability and to capture the necessary variables for each recorded incident, three distinct sources were utilized: the Targeted Automobile Ramming Mass-Casualty Attacks (TARMAC) Attack Database,^17^ the Global Terrorism Database (GTD),^18^ and the vehicle-ramming attack page on Wikipedia.^19^

The TARMAC Attack Database (George Washington University School of Medicine and Health Sciences; Washington, DC USA) collects world-wide incidents caused by MVs from 1973 through 2020 (at the time the data were collected). The GTD (National Consortium for the Studies of Terrorism and Responses to Terrorism [START]) from the University of Maryland (College Park, Maryland USA) collects world-wide intentional incidents caused by different injury mechanisms from the years 1970-2019. The vehicle-ramming attack page from the Wikipedia web site (Wikimedia Foundation; San Fransisco, California USA) collects world-wide intentional incidents caused by MVs around the world, from 1953 through 2021. These resources use open sources and newspaper articles to describe the incidents. The TARMAC Attack Database and Wikipedia collect all the intentional incidents caused by the massive running over of the victims, whether or not they meet the criteria for a terrorist act, as long as there is intentionality, whereas the GTD only records incidents within the context of terrorism.

### Selection of Incidents

All world-wide IMCIs from January 1, 2000 through December 31, 2021 caused by the running over of the casualties using a MV as a weapon were included (MV-IMCIs). An incident was considered an IMCI if there were five or more casualties per incident, as defined by the Spanish and French mean quantitative definition of IMCI.^20,21^ The total number of injured and fatalities recorded for each incident were considered as the number of casualties. A MV was considered a weapon when intentionally used to cause a mass run over, this being the primary traumatic mechanism of the injuries.^1,12^ Incidents where relevant data were missing (distance traveled by the vehicle, vehicle speed, type of vehicle used, and/or number of injuries and/or fatalities generated) were excluded. Those incidents caused by more than one injury mechanism, such as explosives, bladed weapons, or firearms, were also excluded, as well as if casualties were not directly generated by the vehicle. Finally, victims that were not run over, such as a car bomb, a collision with other vehicles with the victims inside, or other circumstances like aircraft incidents, were also excluded.

### Measurements and Outcomes

Variables related to the moment of the incident were collected (place, time, and day of the week of the IMCI); variables related to the vehicle and kinematics (type of MV, vehicle weight in kilograms [kg] obtained from the data sheet of each of the models used in the incident, driving pattern, and average speed (kilometers per hour [km/h]); and variables related to the affected area (street width in meters [m], distance traveled by the vehicle [m], affected area [m^2^], estimation of the density of pedestrians in the affected area [people/m^2^], and the estimated number of people in the affected area) were collected. The Google Maps (Google Inc.; Mountain View, California USA) tool was used to accurately measure the area affected by the vehicle’s path, distinguishing whether the vehicle was occupying the sidewalk or road. To calculate the density of pedestrians in the affected area, the Helbert Jacobs classification of density was used.^22,23^ This classifies density as: fluid, dense, or very dense, granting this quality based on the information present in the sources used, like videos, photographs, and the type of event in which the IMCI occurs. To calculate the estimated number of people in the affected area, the Helbert Jacobs’ formula was used:^22,23^

Estimated number of people = Affected area in m^2^ /density category per m^2^.

### Analysis

The primary outcome variables were the total number of casualties (injured and fatalities) caused by the run over.

Quantitative variables were reported as either the mean and standard deviation (SD) for those that followed a normal distribution, or the median and p25-p75 interquartile range (IQR) otherwise. Qualitative variables were presented as absolute numbers and percentages. Association of the different quantitative variables was assessed using Spearman’s correlation coefficient and simple linear regression. The data were expressed as R^2^ and their corresponding 95% confidence interval (CI). The threshold of statistical significance was established at a value of P <.05. R Studio Team package was used, version 4.2.1 (R Foundation for Statistical Computing; Vienna, Austria.).

The study was conducted in accordance with the Declaration of Helsinki (current version in force, Fortaleza, Brazil, October 2013) and in accordance with the protocol and with the pertinent legal requirements (Law 14/2007 of July 3, on Research Biomedical), and was approved by the Local Ethics Committee of the Hospital Clinic of Barcelona, Spain (HCB/2021/0607).

## Results

**Figure 1.**
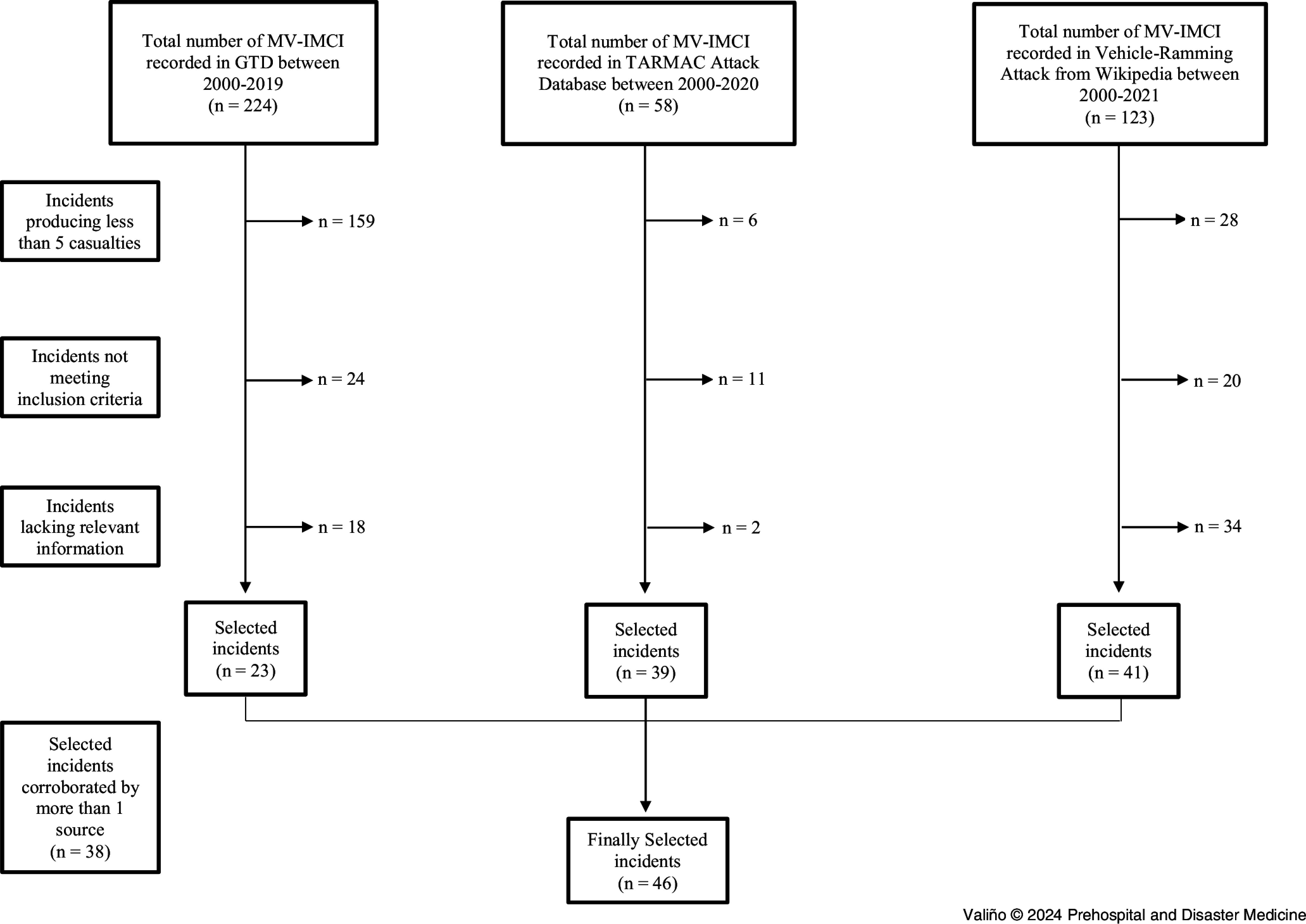
Flowchart of the Selected Incidents from Each of the Data Sources Used. Abbreviations: GTD, Global Terrorism Database; TARMAC, Targeted Automobile Ramming Mass-Casualty Attacks Database; MV-IMCI, motor vehicle intentional mass-casualty incident.

A total of 405 MV-IMCIs from 2000 through 2021 were found in the three sources consulted. After checking the inclusion and exclusion criteria and eliminating duplicates, finally 46 MV-IMCIs were selected. A flowchart of the selection is shown in Figure 1.

**Figure 2.**
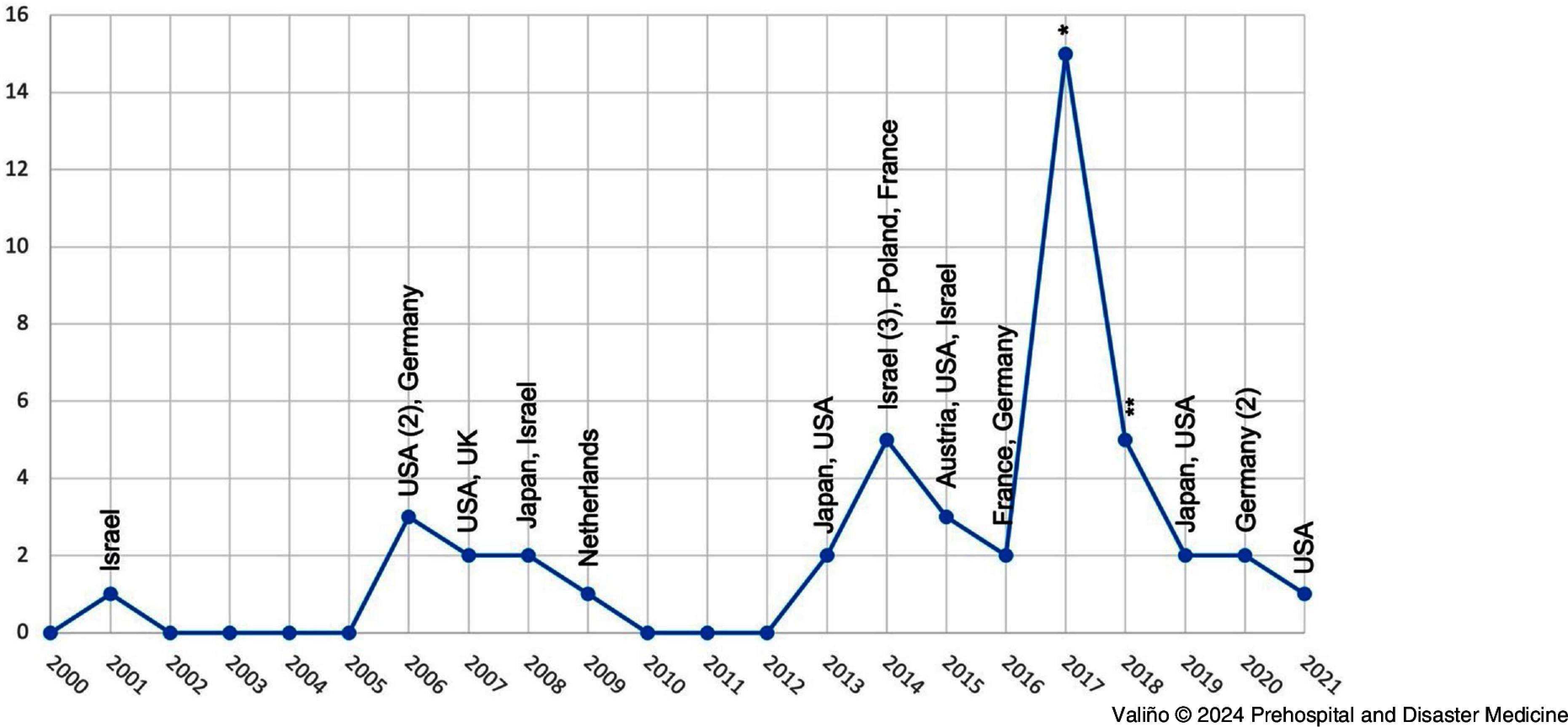
Selection of IMCI Caused by Motor Vehicles from 2000 through 2021, World-Wide. Abbreviations: IMCI, intentional mass-casualty incident; USA, United States of America; UK, United Kingdom. Note: * indicates USA (3), Australia (2), France (2), Spain (2), UK (2), Finland, Sweden, Venezuela, West Bank, and Gaza Strip. ** indicates: Canada, France, Germany, Russia, and UK.

Except for the year 2000 and annual periods of 2002-2005 and 2010-2012 where no MV-IMCIs were observed, between one and 15 incidents were recorded per year, 2017 being the year with the highest number of IMCIs (n = 15). The United States (n = 10), Israel (n = 6), France (n = 5), and Germany (n = 5) were the most affected countries (Figure 2).

The main characteristics of the IMCIs included in the study are summarized in Table 1 and Table 2. They caused a total of 1,636 casualties (1,430 injured and 206 fatalities), with a range of total casualties per incident between five and 544. The proportion of injuries versus fatalities per incident was 89% versus 11% (SD = 0.13), respectively.

**Table 1.** Main Features of MV-IMCIs World-Wide from 2000 through 2021

Characteristics Related to the Used Vehicle and Kinematics		
Type of Vehicle Used by Weight Category^a^	Weight in Kg [Mean (SD)]	Number of Incidents, n (%)
**Category 1**		22 (47.8)
Car	1,377 (SD = 248)	22 (47.8)
**Category 2**		16 (34.8)
SUV	1,944 (SD = 540)	9 (19.6)
Van	1,967 (SD = 468)	7 (15.2)
**Category 3**		8 (17.4)
Truck	18,820 (SD = 14,112)	5 (10.9)
Front Load Vehicle	19,000 (SD = 1,414)	2 (4.3)
Bus	18,444 (SD = 1,233)	1 (2.2)
**Distance Traveled by Vehicles (Meters)** [Median (P25-75)]		130 (16-476)
**Driving Pattern Used**		**Number of Incidents, n (%)**
Acceleration whilst Approaching Target		14 (30.4)
Unknown Pattern		8 (17.4)
Small Group Target		9 (19.6)
Narrow Path with No Escape Route		1 (2.2)
Long Route (>1,000 Meters in Straight Line)		7 (15.2)
Zigzag Driving		7 (15.2)
**Estimated Average Speed (Km/h) Reached by Vehicles Used [Mean (SD)]**		65 km/h (SD = 29)
**Characteristics Related to the Scene**		
Affected Area (m^2^) [Median (P25-75)]		1,591 (180-3328)
**Pedestrian Density Categories (People per m** ^2^ **) According to Jacobs**		**Number of Incidents, n (%)**
Fluid (1 Person/ 0.93m^2^)		28 (60.9)
Dense (1 Person/ 0.42m^2^)		10 (21.7)
Very Dense (1 Person/ 0.23m^2^)		8 (17.4)
**Estimated Number of People in the Affected Area According to Jacobs’ Formula**		2,594 (259-8,028)
**Characteristics Related to Casualties [Median (P25-75)]**		
Total Casualties (Injured and Fatalities)		19 (9-32)
Injured		15 (8-27)
Fatalities		1 (0-4)

Note: Categorical variables are described with absolute frequency (percentage) and quantitative variables with mean (standard deviation [SD]) or median and 25-75 percentiles, as indicated.

Abbreviations: SUV, Sports Utility Vehicle; MV-IMCI, motor vehicle intentional mass-casualty incidents.

a The number of incidents caused in relation to three vehicle categories are described. These categories have been created by calculating the mean weight of each similar vehicle type.

**Table 2.** Range of Casualties in the Selected MV-IMCIs World-Wide (from 2000-2021) According to the Main Variables Studied

Type of Vehicle Used by Category (Weight)^a^		Total Casualties (Injured and Fatalities)	
		Lower-Upper Range^b^	Median (P25-75)^c^
Category 1 (Car)		5-52	14 (8-25)
Category 2 (Van and SUV)		5-115	20 (13-31)
Category 3 (Bus, Truck, and Front Loader)		5-554	27 (16-53)
**Driving Pattern Used**		**Lower-Upper Range** ^b^	**Median (P25-75)** ^c^
Acceleration whilst Approaching Target		6-52	16 (11-25)
Small Group Target		5-21	8 (6-11)
Road Lacked Escape Routes		68	–
Long Route (>1,000 Meters in Straight Line)		16-40	26 (20-37)
Zigzag Driving		5-544	29 (19-92)

**Average Speed in Km/h**	**Number of Incidents, n (%)** ^d^	**Total Casualties (Injured and Fatalities)**	
		**Lower-Upper Range** ^b^	**Median (P25-75)** ^c^
Unknow	6 (13%)	7-14	8 (7-10)
<50	9 (20%)	5-48	11 (8-25)
51-100	28 (61%)	5-544	21 (16-34)
101-130	3 (6%)	40-54	50 (45-52)

**Distance Traveled in Meters**	**Number of Incidents, n (%)** ^d^	**Total Casualties (Injured and Fatalities)**	
		**Lower-Upper Range** ^b^	**Median (P25-75)** ^c^
10-100	21 (46%)	5-52	9 (7-14)
101-500	13 (28%)	6-68	25 (18-48)
501-1,000	5 (11%)	19-115	29 (23-40)
1,001-1,500	5 (11%)	19-68	37 (20-37)
2,240-2,260	2 (4%)	26-544	–

Estimated People On Site (Jacobs’ Formula)	Number of Incidents, n (%)^d^	Total Casualties (Injured and Fatalities)	
		**Lower-Upper Range** ^b^	**Median (P25-75)** ^c^
30-1,000	14 (30.4%)	5-21	10 (6-14)
1,001-3,000	13 (28.3%)	5-54	19 (8-33)
3,001-10,000	10 (22%)	10-48	21 (18-28)
10,001-45,000	8 (17%)	18-115	45 (25-68)
245,717	1 (2%)	554	–

Abbreviations: SUV, Sports Utility Vehicle; MV-IMCI, motor vehicle intentional mass-casualty incidents.

a The number of incidents caused in relation to three categories of vehicle used is described. These categories have been created by calculating the mean weight of each similar vehicle type.

b The lower and upper range of the number of total casualties (injuries and fatalities) found for each of the described categories in the variables analyzed by MV- IMCI.

c Median and 25-75 percentiles of the total casualties produced by each of the categories in the variables analyzed by MV- IMCI.

d Absolute and relative values for each of the categories described in the variables analyzed by MV-IMCI.

Twenty-two percent of the cases occurred on a Saturday, followed by Monday and Wednesday (17.4% each). One-half of the registered MV-IMCIs took place between 12:01am and 6:00pm.

There were three incidents with the minimum number of casualties (n = 5): United Kingdom 2007, Tokyo 2008, and Finland 2017. In these, according to Jacobs, the density category of pedestrians in the affected area was “fluid” with an estimated number of people in the affected area between 127-1,615, the distance traveled by the vehicles ranged between 11 and 41 meters, the weight range of the vehicle was 1,563kg-2,000kg, and the average speed ranged between 48-60km/h.

Forty-five out of 46 IMCIs remained in the range of five to 115 total casualties. The incident that occurred in Nice in 2016 was by far the most damaging, producing 544 total casualties. In that case, the vehicle weighed 18,000kg, zigzagged for 2,240 meters, at an average speed of 90km/h, with a Jacobs’ pedestrian density category of “very dense” and an inferred 245,717 people in the affected area. The second most harmful incident (115 total victims) was in Barcelona in 2017. The vehicle weighed 1,781kg, zigzagged for 700 meters, at an average speed of 80km/h, with a “dense” inference according to Jacobs’ classification and an estimated 32,400 people in the affected area.

Nearly one-half of the MV-IMCIs were caused by cars. Sports utility vehicles (SUVs) and vans were the second most used group of vehicles, accounting for one-third of the cases. The least utilized vehicles were the heaviest ones (18,000kg-20,000kg), making up for only 17.4% of the incidents. These also presented the widest range of total casualties (five to 544) among incidents.

The driving pattern was known in 83% of the analyzed MV-IMCIs. Acceleration whilst approaching the target was the most frequent (almost one-third of all incidents), whereas long and zigzag routes were associated with the highest number of casualties; moreover, when the vehicle traveled over 1,000 meters, the lowest number of casualties (n = 16) was higher compared to the other driving patterns (five to six). Notably, there was only one instance where the road lacked escape routes, resulting in 68 victims (Berlin 2016).

In the 40 MV-IMCIs (87%) where the vehicle average speed was known, it ranged from four to 130km/h. Only nine of them presented an average speed below 48km/h, while the remaining 31 incidents had vehicles with an average speed equal to or exceeding 60km/h. Three incidents recorded an average speed exceeding 100km/h, resulting in total casualties ranging from 40 to 54 (Washington [USA] 2007, Oklahoma [USA] 2015, and London [UK] 2017).

Regarding the distance traveled by the vehicles, a range between 10 meters (reported in 10 MV-IMCIs) and 2,260 meters (Toronto 2018) was found. In almost one-half of the registered MV-IMCIs, the vehicles traveled less than 70 meters and in 74% of the MV-IMCIs, the distance was below 500 meters. Among the 12 (26.1%) incidents where the vehicle traveled a distance greater than 500 meters, the lowest number of casualties was 19. In two incidents, Toronto and Nice, the vehicle traveled over 2,000 meters, resulting in 26 and 544 total casualties, respectively.

The estimation of the number of people in the affected area of the MV-IMCI, using the Jacobs’ formula, resulted in a range from 36 to 245,717 pedestrians. In seven out of the 46 (15.2%) registered MV-IMCIs, the estimate was less than 100 people. Notably, incidents with an estimate of over 3,000 people on the route resulted in a minimum of 10 total casualties, while estimates exceeding 10,000 people were associated with a minimum of 18 casualties. The MV-IMCI with the highest estimation was, as previously noted, Nice in 2016 with 245,717 people, and it also corresponded to the incident with the highest number of total casualties, reaching 544. In the remaining 45 incidents, the estimated number of people did not exceed 45,000.

**Figure 3.**
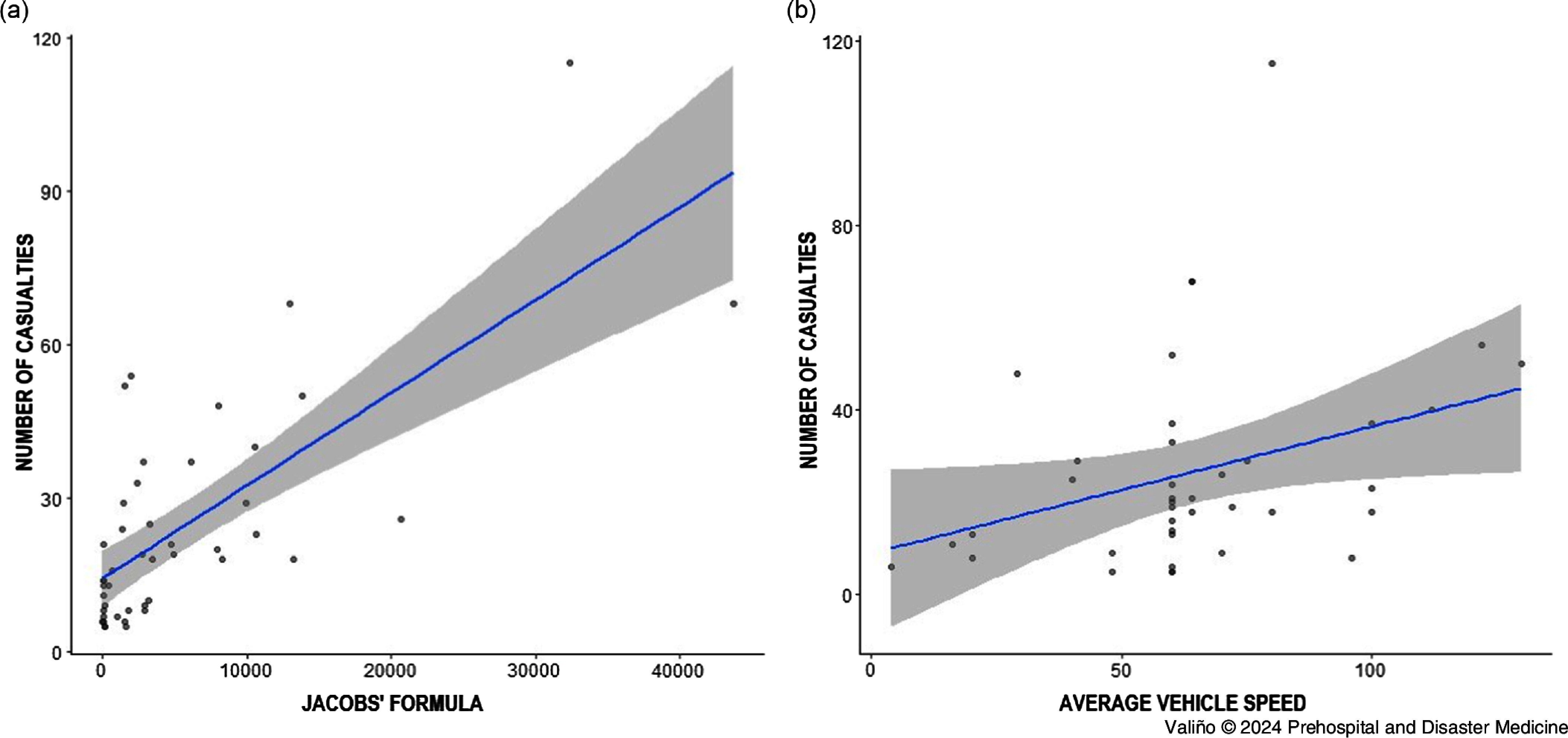
Linear Regression Model of the Two Main Variables that Showed a Significant Association with the Number of Casualties: **(a)** Estimated Number of People On Site (Jacobs’ Formula); and **(b)** Average Vehicle Speed.

Statistical analysis, as shown in Table 3, exposed a significant positive association of the total number of casualties with the affected area (R^2^ = 0.57), the distance traveled (R^2^ = 0.59), the average vehicle speed (R^2^ = 0.42), the Jacobs’ category density (R^2^ = 0.39), and the estimation number of people in the affected area by Jacobs’ formula (R^2^ = 0.64). The vehicle weight did not reach statistical significance (P = .065). Figure 3 shows graphical representation of the linear regression model of the two main variables that showed a significant association with the number of casualties.

**Table 3.** Correlation Analysis between the Different Independent Variables and the Number of Casualties Produced by MV-IMCIs World-Wide from 2000-2021.

Independent Variables Analyzed	R^2^ ^a^	95% CI^b^	P Value
Affected Area (m^2^)	0.57	0.55-0.59	<.001
Distance Traveled (m)	0.59	0.56-0.62	<.001
Vehicle Weight (Kg)	0.18	0.17-0.19	.065
Average Vehicle Speed (Km/h)	0.42	0.40-0.44	.004
Pedestrians’ Density Categories (People per m^2^) According to Jacobs	0.39	0.37-0.41	.003
Estimated Number of People On Site (Jacobs’ Formula)	0.64	0.61-0.67	<.001

Abbreviation: MV-IMCI, motor vehicle intentional mass-casualty incidents.

a Coefficient of determination.

b 95% Confidence Intervals.

## Discussion

The present study identifies the variables that have a significant association with the number of casualties (injured and fatalities) produced in a MV-IMCI. It also gathers, shows, and analyzes the most relevant information about MV-IMCIs in the recent literature. As far as can be determined, this study is the first to demonstrate a positive association between specific variables and the number of casualties in such incidents, in addition to assessing the influence of their respective weights on the overall outcome. This can be very valuable information to estimate the number of total expected casualties in the initial phase of an IMCI of these characteristics, especially useful to scale and adjust the health care response resources.

Both under-preparedness and over-response in the face of an IMCI are deleterious. For example, the first assessment made by the EMS in the MV-IMCI in Berlin (2016) under-estimated the number of casualties by 50% of the real number.^6,9^ But since the hospitals that responded to the incident did not have precise information on the approximate number of victims, some, such as the Bundeswehr Hospital, over-prepared: it activated its emergency plan at its maximum level, but it only received four patients (three red category and one yellow).^6,9^

The estimated number of people in the affected area (Jacobs’ formula) and the achieved average speed are the variables that exhibit the most robust association with the expected number of casualties. Incidents with estimated presence of over 3,000, 10,000, and 20,000 individuals along the vehicle’s route yielded a minimum of 10, 18, and 26 casualties, respectively. Conversely, when average speeds were below 50km/h, the casualty count did not exceed 48 people, whereas in incidents where the vehicle exceeded 100km/h, the range of casualties ranged from 40 to 54. This limited increase in victims at higher speeds may be attributed to the reduced feasibility of a zigzag route and diminished precision in targeting, factors that can increase the casualties.

Although previous studies had assumed that the weight and type of vehicle used could have a correlation with the number of casualties caused,^6,7,14,24^ these variables weren’t significantly associated in the present study. Seven out of eight large-tonnage MV-IMCIs included in the study yielded a maximum of 68 victims per incident, and two of them had a victim count not surpassing six. However, the Nice incident presented a very significant number of casualties (n = 544), which has exerted a substantial influence on the results. It has to be noted that in this incident, there was also a combination of a large estimated number of people in the affected area (n = 245,717) and a considerable average speed attained by the vehicle (90km/h), which could have influenced in number of victims rather than its weight.

The findings obtained from the study have implications for estimating the scale of both prehospital and hospital responses during the early phase of the MV-IMCI. Activating the hospital emergency plan requires increasing surge capacity and reorganizing resources despite limited information.^6,7,25,26^ By considering the affected area and population density, a preliminary minimum and maximum number of casualties involved in the incident can be calculated using Jacobs’ formula. This information is easy to obtain by emergency coordination centers, either by cameras in public places that can provide images instantly (Barcelona 2017), or by the possibility of interrogating the alerters/notifiers/informers. The place, time, and type of event will also help to estimate the density. Moreover, if the average speed of the vehicle is known, this estimation can be further refined. Consequently, a range of casualties can be estimated (Table 2) that require immediate attention until more information becomes available and can perform the first triage of the victims, especially considering that there can be potential delays in conducting the triage due to security concerns within the area.^6,27–29^ Even then, this information can continue to be useful, as a significant proportion of IMCI patients arrive at hospitals independently, from 10 minutes to within the first hour of the incident, without prior alert or prehospital triage,^6,15,27,30^ significantly impacting hospital mortality in these cases.^3,15^

In addition to estimating the range of casualties (Table 2), it may be useful to gather data on injuries as well as the requirements for intensive care units (ICUs) and operating rooms (ORs) in order to determine the extent of care in the hospital phase.

Thus, different studies have shown that traumatic brain injury is the main injury in serious patients from MV-IMCI, being present in 36%-63% of these patients. Of the total number of patients injured, 15%-23% will be serious, 23%-30% will require admission to the ICU, and 33%-47% will require surgery in the OR.^7,10,14,15^

## Limitations

The study at hand is subject to several limitations. The primary one is the limited availability of incidents with the complete set of variables required to make an accurate estimate of the number of casualties in the initial stages of the MV-IMCI. Consequently, the sample size was restricted to 46 cases, potentially introducing selection bias. However, with the inclusion of intentional incidents outside the terrorist context and diverse information sources can help to mitigate this bias. Additionally, it is important to note that most of the recorded data originate from written press sources. To minimize potential information bias, data comparisons were conducted across sources and referenced scientific journals when available for data validation. Finally, the data analyzed do not allow for an exact number of casualties, but rather an estimate with a range. Nevertheless, the information provided may be useful at a time when available data are scarce. Future efforts will aim to collect a larger number of incidents in order to design a predictive model for more accurate early estimation of the casualties.

## Conclusion

The estimated number of people in the affected area and vehicle’s average speed are the most significant variables associated with the number of casualties in MV-IMCIs. These findings facilitate an initial minimum and maximum estimation of the number of casualties during the early stages of the incident. Further research is warranted to develop a more robust and user-friendly formula within a predictive model design. Such efforts will aid to improve the early scaling of the health care response to effectively address incidents of this nature.
